# Supplementary material for: Crosstalk between Nutrition, Insulin, Juvenile Hormone, and Ecdysteroid Signaling in the Classical Insect Model, Rhodnius prolixus
Source: Int J Mol Sci. 2022 Dec 20;24(1):7. doi: 10.3390/ijms24010007 (PMC9819625; doi:10.3390/ijms24010007)
Supplement: Supplementary file 1 [file ijms-24-00007-s001.zip › ijms-2097999-supplementary.pdf]

Supporting Information for

Crosstalk between Nutrition, Insulin, Juvenile Hormone, and Ecdysteroid Signaling in the Classical Insect Model, *Rhodnius prolixus*

Jimena Leyria [1,*], Samiha Benrabaa [1], Marcela Nouzova [2], Fernando G. Noriega [3,4], Lilian Valadares Tose [5], Francisco Fernandez-Lima [5], Ian Orchard [1] and Angela B. Lange [1]

[1] Department of Biology, University of Toronto Mississauga, Mississauga, ON L5L 1C6, Canada
[2] Biology Center of the Academy of Sciences of the Czech Republic, Institute of Parasitology, 37005 České Budějovice, Czech Republic
[3] Department of Biological Sciences and Biomolecular Science Institute, Florida International University, Miami, FL 33199, USA
[4] Department of Parasitology, University of South Bohemia, 37005 České Budějovice, Czech Republic.
[5] Department of Chemistry and Biochemistry and Biomolecular Science Institute, Florida International University, Miami, FL 33199, USA
* Correspondence: jimenal.leyria@utoronto.ca; Tel.: +1-905-569-4752

This file includes:

Figure S1

Table S1

A

Component	RT (min)
JH I	9.824
JH II	9.623
JH III	9.415
JHB$_3$	8.623
JHSB$_3$	8.951
Methyl Farnesoate	10.168

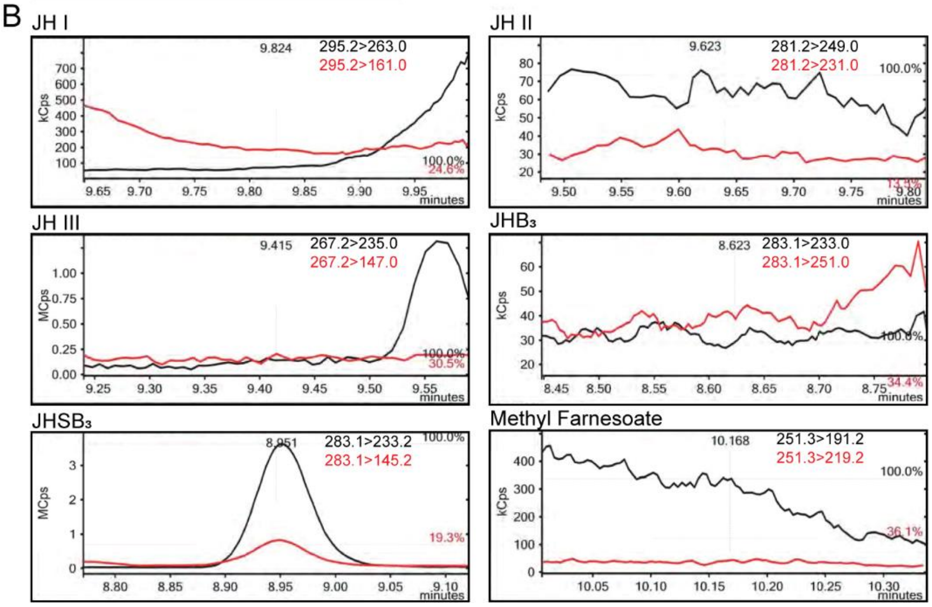

Supplementary Figure S1. Identification of JHSB$_3$ from the hemolymph of *R. prolixus* adult females. (A) Retention times of each component are shown in minutes. (B) Typical LC-MS/MS peaks of JH homologs and MF. It shows the relationships between the retention times in minutes (x-axis) and the signal intensity (cps; counts per second) (y-axis). Black lines represent the signal intensity of the primary transition, and red lines represent the intensity of the secondary transition. Only JHSB$_3$ shows the presence of the right product ions at the correct retention time.

Supplementary Table S1. Primers used for qPCR.

Gene code	Primers to qPCR	Sequence (5→3)
RPRC013511	Vg 1_forward	TTGCTAGTCGCATGAACCTG
	Vg 1 _reverse	TTTAGTGGTGCATCGCTCTG
RPRC009875	Actin_forward	AGAGAAAAGATGACGCAGATAATGT
	Actin _reverse	ATATCCCTAACAATTTCACGTTCG
RPRC014419	Rp49_forward	GTGAAACTCAGGAGAAATTGGC
	Rp49_reverse	AGGACACACCATGCGCTATC
RPRC007496	Thiol_forward	CAAAGTTAATGTACACGGTGGTG
	Thiol_reverse	CTCCAGACTTCAACGCTGTTA
RPRC007884	§HMGS_forward	GCAACTGTTTGAAGAAAGTGGTA
	HMGS_reverse	AAGCACTGGTACCTCCAAAG
Supercontig RproC3:KQ03422 6 minus strand 878233-875494	§HMGR_forward	GGCATAGAAAGAAGATGACCAAAC
	HMGR_reverse	GCACGAGTATCAAGACAACAATATG
RPRC014277	§MEVK_forward	GAAAGATCAAGAGGAACGAGGAG
	MEVK_reverse	CGCTTATGTGAGACACCTAATGAT
RPRC006212	§P-MEVK_forward	AAATCGTTTCTGACGAACAAGTG
	P-MEVK_reverse	GCAATGACAACATCCCATTCAG
RPRC012093	§PP-MEVK_forward	CGTGGCCTTCCAGTTCAA
	PP-MEVK_reverse	GTATTTGAGGAGACCAGGTTCG
RPRC003718	§IPPI_forward	CACCAATTACGCCTTGGTTTAG
	IPPI_reverse	GTGGATATTCACGTGGTCTTGA
RPRC010547	§FOLD_forward	AAACCGAGCGATGTTGT
	FOLD_reverse	GTAGGTTGGATAACTAGTTCTGAT
RPRC002910	§FALDH_forward	AGTACCTTACAGTCTAGTATTTGCC
	FALDH_reverse	GATCTGTCTTCAGCACCGTT
RPRC011659	§JHAMT_forward	GGACCAGGCGATGTTACTTT
	JHAMT_reverse	CCAAATCATCAGAAATATCGCTTCC
RPRC000513	§Epox_forward	CGGAGAATTGATTCATGATGATTGG
	Epox_reverse	GTAACGGCGGTGACAGTAAA
RPRC014226	§FPPS_forward	CGCAGTAGTTGCAATGCATAAAG
	FPPS_reverse	GCTTCTTGACAGCGGCTATT
RPRC004412	§FPPP_forward	GCTTGAATCCTAGAAGAGCGTTA
	FPPP_reverse	ACCGGTAAGTACAAGCAATGTAT
RPRC011241	*Spook_forward	TGGCATTCTCCGATTGGTCT
	Spook_reverse	TCATTGAGCAACGTGTCCAGT
RPRC006417	*Shadow_forward	GAGGCAAGTTTTCGAAGTGG
	Shadow_reverse	TGATTCATAATTCGGCGATG
RPRC011595	*Dib_forward	TTGCACACTACCGTTTGTCG
	Dib_reverse	AATGCGAGCAAGTGGTTTTT
RPRC009372	*Phantom_forward	TGCCATACACGGAAGCATGT
	Phantom_reverse	CGATAGCCTGCCAGTTCAGT

RPRC006945	*Shade_forward	ATTCTTTGGGCTCCCATTCT
	Shade_reverse	GCCATGAACACACTTTGCAC
RPRC001631	*Nvd_forward	TGCTCCACTCATCTTGAAAGC
	Nvd_reverse	ACTTGGCAATGGTTTTGTCTGT
RPRC006251	Rhopr-IR1_forward	TGGTTCGGTGGGAGACAGCT
	Rhopr-IR1_reverse	AGAAGTCGAGCGACACCAGT
GECK01011918.1	Rhopr-IR2_forward	CTTGCTGGGGTTGGAGAATA
	Rhopr-IR2_reverse	TCAGACTGACGACGGAAGTG
KF740716	AT-R_forward	TGCCGAACGTCATTACACCA
	AT-R_reverse	ATGGCCAGGTATGTTGTCCG
RPRC004708	ASTA-R_forward	TTGTCGTAGCGGTCAACCAA
	ASTA-R_reverse	AGGTGTCACCAAAAGGCCAA

§Primers reported by Villalobos-Sambucaro et al. [50]; *Primers reported by Benrabaa et al. [52]
